# Quality of Life in Children with Developmental Language Disorder

**DOI:** 10.3390/children13030418

**Published:** 2026-03-19

**Authors:** Mélanie van Barreveld, Iris Duinmeijer, Annette Scheper, Britt Hakvoort, Constance Vissers

**Affiliations:** 1 Research Department, Royal Kentalis, 3527 JP Utrecht, The Netherlands; 2 Behavioural Science Institute, Radboud University, 6525 GD Nijmegen, The Netherlands; 3 Research and Development Department, Nederlandse Stichting voor het Dove en Slechthorende Kind (NSDSK), 1073 GX Amsterdam, The Netherlands; 4 Research Department, Royal Dutch Auris Group, 3031 RT Rotterdam, The Netherlands

**Keywords:** developmental language disorder, quality of life, social–emotional functioning, development, behaviour, well-being

## Abstract

**Highlights:**

**What are the main findings?**

- The quality of life of children with DLD is below that of their typically developing peers at 4 and 9 years.
- Emotional well-being is particularly vulnerable in children with DLD.

**What are the implications of the main findings?**

- Quality of life could be an additional outcome to monitor in children with DLD.
- Early peer problems are a potential indicator of reduced quality of life.

**Abstract:**

**Background/Objectives:** Developmental language disorder (DLD) has widespread and persistent consequences for children’s development, extending beyond language. Quality of life (QoL) can also be affected, potentially related to difficulties with cognition (e.g., language ability), behaviour (e.g., social–emotional functioning), and/or environmental factors (e.g., multilingualism). This study set out to characterise changes in the QoL of children with DLD and to identify related factors. **Methods:** Data were collected at 4 and 9 years from children who had attended early language intervention groups before age 4. Parents completed online questionnaires, including the KINDL for QoL. The KINDL measures QoL on six domains: physical well-being, emotional well-being, self-esteem, family well-being, social well-being, and school functioning. Structural Equation Modelling (SEM) was used to assess the relationship between change in QoL and cognitive, behavioural, and environmental factors. **Results:** At both time points, the QoL of children with DLD was reduced compared to the normative group on all domains except self-esteem and family. Emotional well-being and self-esteem scores were significantly lower at 9 years compared to 4 years. Peer problems and multilingualism emerged as influential factors regarding changes in QoL over time. **Conclusions:** The QoL of children with DLD is vulnerable between age 4 and 9. Overall, QoL might deteriorate in this period, specifically emotional well-being. Early peer problems and multilingual status influence the changes observed in different aspects of QoL, although these effects should be interpreted with caution.

## 1. Introduction

The term developmental language disorder (DLD) has been suggested to best capture the heterogeneous group of children who experience problems with language acquisition and development in the absence of any neurological or perceptual impairments [1]. Children who encounter challenges acquiring language from a young age face an increased risk of problems in their social, emotional, and academic development [2,3]. Evidence indicates that these difficulties often persist into adolescence and adulthood [4,5,6]. Recent research has also demonstrated links between low language abilities and quality of life (QoL) (for a review, see [7]). Collectively, these findings underscore the significant impact of DLD on daily functioning.

QoL is conceptualised as an individual’s or a parent/teacher’s perspective on this individual’s well-being in different life areas, such as the family and school setting. In recent studies on DLD and related developmental disorders such as ADHD, Autism Spectrum Disorder (ASD), and epilepsy, QoL has been referred to as one’s perception of their physical, psychological, and social functioning beyond the disorder’s symptomatology [8,9]. As a result of the multidimensionality of QoL, outcome measures may differ with regard to the items they include to evaluate (aspects of) QoL.

Given that QoL relates to various aspects of life across life stages, it is crucial to understand if and why it changes. Monitoring changes in the QoL of children with DLD might be important for both clinical practice and the educational sector [7]. Therefore, the present study explores QoL in a group of Dutch children with DLD. In the Netherlands, young children (at least up to the age of 3) receive the label “presumed DLD”. DLD can be confirmed after this age. The criteria for presumed DLD are identical to those for DLD [10]. The current research studied the changes in QoL and explored potential protective factors of QoL in this population.

### 1.1. Quality of Life in DLD

Research on the QoL of populations with (chronic) health conditions or disorders, such as cancer, mental illness, or COVID-19 disease, is abundant (for a review on QoL research in medicine, see [11]; for COVID-19, see [12,13]). However, studies specifically focusing on the QoL of individuals with neurodevelopmental disorders such as ASD and DLD have been notably less common, particularly longitudinal studies [7]. Evidence suggests that QoL is affected in individuals with DLD compared to children with typical development (TD) across ages ranging from childhood to adulthood [5,6]. For instance, the physical and psychological well-being, social acceptance, and quality of parent relations of 10-year-old children with DLD are below those of TD children [14]. A study by [8], based on longitudinal data from 70 Australian school-aged children with DLD (minimally 1.25 SD below the mean on standardised language test(s)), adds important information on developmental patterns. Using the Pediatric Quality of Life Inventory (PedsQL), they investigated, amongst others, the effects of standardized language measures and behavioural problems on QoL. Visual inspection of the QoL scores at 4, 7, and 9 years suggests that the QoL of children with DLD deteriorates during this period. This trend is also present in the TD group (*n* = 802), but to a lesser extent. Using data from the same cohort [15] aimed to explore the associations between language problems and QoL and to identify trajectories of QoL development between the ages of 4 and 13 years. They maintained broader inclusion criteria than [8] by including participants with comorbid conditions, and named their language-impaired sample “low language” (LL, minimally 1.25 SD below the mean on standardised language tests). The number of participants differed per measurement, but the TD group ranged between 674 (age 9) and 1322 children, and the low language group ranged between 33 (age 13) and 160 children. They report that the QoL of children with LL is less likely to remain stable or increase over time than that of children with TD. The authors did not report on other factors besides language that define participants in each trajectory.

### 1.2. Factors Associated with QoL in Children with DLD

Timely detection of lowered QoL in DLD calls for the identification of factors influencing (changes in) QoL. This is not an easy task, given that myriad factors can impact an individual’s QoL, irrespective of health conditions. From a neuropsychological perspective, the problems observed in children with DLD can be understood as arising from the interplay between cognition, behaviour, and child-specific environmental factors [16,17,18]. These factors (cognition, behaviour, and the environment) are proposed to be interconnected. Changes or impairments in one area are hypothesised to impact both or either of the others [16,17,19]. QoL, then, is the result of the interplay between cognition, behaviour, and environment. When studying QoL, it is thus necessary to examine these different factors in relation to QoL.

*Cognitive factors* encompass those factors related to abilities such as language and executive functions (EF). The differences in QoL between DLD and TD might seem to confirm an effect of language difficulties on QoL, but research exploring the impact of language abilities on QoL in DLD suggests that the severity of the language problems explains only part of the variation found in the QoL [8,20]. Studies in [8,21] examined QoL among children with severe DLD. Notably, Nicola and Watter report lower QoL scores despite applying a less stringent criterion for language scores (>1.5 SD below the mean compared to Eadie et al.’s > 2 SD below the mean for the severe DLD group). Another cognitive factor that may relate to QoL in children with DLD is EF. The link between DLD and impairments in EF is well-established for both preschool and school-aged children [19,22]. While the connection between QoL and EF has not been explored in children with language difficulties, in the literature, there exists research on this relationship in other neurodevelopmental disorders (ASD: [23]; ADHD: [24]).

Secondly, problems in *behaviour*, such as difficulties in communicative participation (CP) or social–emotional functioning (SEF), might also affect QoL. CP is a functional measure of language behaviour, a concept that has garnered increasing attention in recent years. More specifically, it is the ability to understand others and be understood in a social context [25]. A recent study by [26], utilising data from the same cohort as the present study, found that CP plays a mediating role between standardised language measures and well-being in Dutch children with DLD. In the area of SEF, children with DLD might experience difficulties with peer relations, higher levels of hyperactivity, and emotional problems such as anxiety or emotional dysregulation (e.g., [27,28,29,30]). QoL measures often tap into SEF as they include questions about the experience of emotions (basic feelings of, e.g., anger, sadness, worry) and social relations (e.g., being liked by others, getting along with others). Measures of SEF can therefore be expected to correlate with or predict QoL [26]. The emotional and peer relations scales from the Strengths and Difficulties Questionnaire (SDQ, ref. [31]), a screening for behavioural problems, have been found to partially predict QoL at 9 years [8].

A final set of influential factors on QoL are those that are *child-specific* or *environmental*. These factors include, but are not limited to, socio-economic status (SES), gender, multilingual status, and access to special education or support. SES is often associated with QoL [32,33], as is also true for male gender [8]. Additionally, attitudes and values regarding (in)appropriate behaviour (e.g., emotions and expression thereof) may be influenced by the culture in which one is raised ([34], p. 2). Given that multilingualism typically entails growing up in a culture different from that of monolingual speakers living in the same country, multilingual status might relate to SEF and, consequently, QoL in ways that differ from monolingual experiences. Some studies indicate that children who are balanced bilinguals or are proficient in the societal language may experience levels of well-being that are similar to, or higher than, those of monolingual speakers of that language [35,36]. As such, a multilingual upbringing can shape QoL in both positive and negative ways, at least in TD children. Finally, access to support services is marked by both limited availability and unclear effects on development beyond language abilities. Ref. [37] demonstrate that many Canadian and Australian children in need of speech–language therapy or related services fail to receive or utilise these services. The potential consequences of using such services for well-being are unclear and could go in two directions. One possibility is that, considering that specialised services (e.g., treatment or special education) are aimed at increasing communicative participation, children’s well-being will also benefit from these services. Alternatively, ref. [14] reported lower scores for school functioning and social support among children with language difficulties who received (psycho)therapy or attended special school or educational programs. In light of these findings, the educational system in the Netherlands is particularly relevant. Here, children with DLD may qualify for special education for children with DLD if they meet specific criteria, including scores below −1.5 SD below the mean on language tests, no hearing problems, a nonverbal IQ above 70, and significant communication challenges that hinder their ability to benefit from regular education. While both special and mainstream schools aim for the same educational outcomes, special schools offer a curriculum adjusted to individual needs [38]. Speech–language therapy (SLT) is provided at school, either individually or in small groups, and class sizes are smaller. Children with milder communication or language problems might be eligible for extra support within mainstream schools through collaboration with specialised centres, while some may attend mainstream education without any support. Notably, it remains unclear whether the options confer benefits over one another [39], a question to which no answer may exist due to a complex interplay of influential factors.

### 1.3. The Present Study

Knowledge of QoL can guide decisions on therapy and other forms of support offered to children with DLD. Neurodevelopmental disorders may manifest differently over time (i.e., growing into deficit principle), underscoring the importance of mapping development over time and the early identification and monitoring of predictive factors. Existing literature on QoL in children with language difficulties has laid the ground for further research, as these studies have identified (1) factors that may relate to later QoL, and (2) identified trajectories of QoL in this population [8,15]. A more integrated account of changes in QoL and the variables related to this has not yet been carried out. This study attempts to contribute to the current literature on QoL in DLD by focusing on changes in the QoL of school-aged children with DLD and factors that drive such changes. QoL is a multifactorial construct that integrates elements from cognition, behaviour, and the child’s environment cohesively. To comprehend the QoL of children with DLD, it is essential to consider all these domains in relation to QoL development.

Therefore, this study was guided by the following questions:

1. How does QoL change in children with DLD between 4 and 9 years?
2. What is the role of cognitive, behavioural, and/or environmental factors in the change in QoL between 4 and 9 years in this group?

The present study reports on a longitudinal cohort which had a diagnosis of (presumed) DLD at the time of recruitment (see Section 2.1 Participants below). Therefore, we also exploratively investigated the two research questions in a subgroup of children whose DLD diagnosis was confirmed at a later age.

## 2. Methods & Materials

### 2.1. Participants

The present study draws on data collected by a larger research study called the Dutch Longitudinal DLD study (DLDLD study, or in Dutch, “Taal in Zicht” (more information on Taal in Zicht can be found on the following page: For researchers—Project Taal in zicht, https://www.projecttaalinzicht.nl/for-researchers/ (accessed on 15 December 2025))). This longitudinal study follows the development of a large group of children who were diagnosed with (presumed) DLD. Due to early screening procedures in the Netherlands, DLD can often be identified early in life, but up to (at least) 3 years of age, the term “presumed” DLD is used. As mentioned previously, the criteria for this label are identical to those for DLD. Children’s language abilities are assessed with the Schlichting tests for Language Comprehension and Production and the Peabody Picture Vocabulary Test (PPVT). Children are diagnosed with (presumed) DLD when their score on any of these tests falls 1.5 SD or more below the norm. Further criteria include a nonverbal IQ ≥ 70, no hearing problems, and no other biomedical condition(s).

The DLDLD study commenced in 2015 and recruited 600 children at early language intervention groups located across the Netherlands. See [26] for a description of the intervention groups in the Netherlands. Data is being collected at seven time points (T0–T6) between the ages of 4 and 24 years, three of which have already been completed. T0 took place at the conclusion of the language intervention group (4 years), T1 was 6 months later, and T2 corresponds to grade 3 (age 6–7 years). Currently, T3 is in progress, occurring in grade 5 (8–9 years). At T4, participants will be 13 years old, at T5, they will be 19 years old, and the final time point will take place at age 24. The current study reports on the subgroup of children who completed the QoL measure at T3 (*n* = 312). Sixteen participants were excluded because they were outliers on age at one of the two measurements for QoL used in this study, resulting in a sample of 296 children included in this study.

Since children were labelled “presumed DLD” at the start of the study, the DLD diagnosis was often confirmed at a later point. Information gathered on official diagnosis and type of education was combined to determine which participants had and which participants did not have what we will call “confirmed” DLD. Children were identified as having confirmed DLD if:

1. Parents indicated DLD was diagnosed after children left the early intervention group; or,
2. Children attended special education for DLD after leaving the intervention group (In the Netherlands, children are only eligible to attend special education for DLD when they meet the criteria for DLD.)

Table 1 presents the characteristics of the total sample and of the subgroup with confirmed DLD. Note that some participants have incomplete data on one or more of the characteristics. Participants’ socio-economic status (SES) was based on the educational attainment of the highest-educated parent. High SES included parents who attended higher education, and medium SES was attributed to those with a vocational education and training (VET; *MBO* in Dutch). Low SES was assigned to children whose parents’ educational attainment was high school or below.

### 2.2. Measures

#### 2.2.1. Quality of Life

QoL was assessed using the Dutch translation of the Kinder Lebensqualität fragebogen (KINDL) questionnaire. Translations adhered to the publishers’ protocol and were conducted by the project group of the DLDLD study. At T1, parents completed the KINDL-R(evised) for children aged 3 to 6 years (Kiddy-KINDL 3–6 y [40]). At T3, parents completed the version for children aged 7 to 13 years old (Kid-KINDL 7–13 y [40]). Both versions consist of six subscales, namely physical well-being, emotional well-being, self-esteem, family, friends (from hereon: social well-being), and school functioning. Each scale consists of 4 items, meaning the questionnaire consists of 24 items in total. Items were rated on a 5-point Likert scale and total scores ranged from 0 to 100, with higher scores indicating better QoL. German normative means and standard deviations were available for all parent-rated questionnaires [41]. Psychometric analyses were run with all available data. At T1 and T3, internal consistency was above *α* = 0.60 on all subscales and *α* = 0.85 for the T1 total QoL score and *α* = 0.89 for the T3 total QoL score.

#### 2.2.2. Language Ability

At T0, children’s language comprehension and production were assessed with the Peabody Picture Vocabulary Test (PPVT-III-NL [42]), the Schlichting Test for Language Comprehension, and the Schlichting Test for Language Production [43,44]. The PPVT is a picture-matching task that evaluates word comprehension. The Schlichting Test for Language Comprehension includes object identification and sentence comprehension tasks. The production test assessed expressive vocabulary and expressive grammar through naming and sentence elicitation tasks. The raw scores for language comprehension, expressive vocabulary, and expressive grammar were converted to quotient scores with a mean of 100. Dutch normative scores were available for all tests.

#### 2.2.3. Communicative Participation (CP)

Communicative participation, a functional measure of language abilities, was assessed at T1 through a questionnaire with statements inquiring about communication with a different type of communicative partner (an extension of a short questionnaire used by [45]). An example of an item is *“My child is able to communicate independently with peers”.* Parents rated their child’s communicative participation on a 10-centimetre visual analogy scale (VAS). The full questionnaire is supplied in the Supplementary Materials. The questionnaire initially consisted of nine items, but two items had to be removed due to low internal consistency. As such, the total score has a maximum of 70, with a Cronbach’s alpha of 0.88 for reliability.

#### 2.2.4. Peer and Emotional Problems

The peer problem and emotional problem scales from the Strengths and Difficulties Questionnaire (SDQ; [31], Dutch version: [46]) characterised the children’s peer relations and abilities, as well as their emotional difficulties. Parents completed the SDQ at T1. The SDQ contains 25 items in total, divided into five subscales: peer problems, emotional symptoms, conduct behaviour, inattention/hyperactivity, and prosocial behaviour. Items were rated on a 3-point Likert scale with the options not true (0 points), somewhat true (1 point), or certainly true (2 points). The maximum score for each subscale of the SDQ is 10 points. Internal consistency and construct validity were measured using all available data. Internal consistency was *α* = 0.343 and *α* = 0.645 for peer and emotional problems, respectively.

### 2.3. Procedures

Data were collected at multiple timepoints (see Table 2). As this study utilises a subset of the data from the DLDLD study, only information on the measures relevant to the current analyses is presented.

At T0, language ability was tested by a speech–language therapist or clinical linguist before children left the intervention group. At T1, which was six to nine months after leaving the intervention group, parents completed the measures for communicative participation, peer problems, and quality of life through an online Routine Outcome Monitoring (ROM) platform. This also included a background questionnaire addressing, amongst others, educational support, SES, and multilingualism. At T3, parents received another ROM invitation containing the QoL questionnaire and a background questionnaire that asked about their child’s DLD diagnosis.

Data collection for T3 is ongoing, leaving attrition unknown at this moment. The analyses in this paper include only participants for whom the KINDL was completed at T3. Missing data on other variables was allowed. Data are considered missing when parents did not respond to reminders via e-mail or phone calls.

### 2.4. Statistical Analysis

Analyses were pre-registered on the Open Science Framework (https://osf.io/2vcsq/overview?view_only=f299a2fef2314b98a7e1642df32a38ef, accessed on 4 March 2026). Diversions from the preregistration will be mentioned where applicable.

All analyses were run in R 4.4.2 and were subject to a Benjamini–Hochberg correction for multiple testing. Analyses were run for the total KINDL score as well as the scores on the six QoL subscales (i.e., physical well-being, emotional well-being, self-esteem, family, social well-being, and school). Scores were sums of items corresponding with each scale, and the total KINDL score was a composite of all items. Data were checked for normality and outliers before analysis.

To examine changes in QoL between T1 (baseline) and T3 (follow-up), paired samples *t*-tests were conducted. Only participants with QoL data at both measurements were included in the analyses (*n* = 291). The significance level was set at 0.05.

To answer the second research question, Structural Equation Modelling (SEM) was used (*lavaan* package, version 0.6-21). The models were estimated using robust maximum likelihood (MLR), which gives statistics robust to non-normality. Missing data were handled using full information maximum likelihood (FIML), which uses all available data to fill in missing values under the assumption of missing at random. Data in our dataset were missing due to language tests not being administered in the intervention groups, or because parents did not complete (one of the) online questionnaire(s). Little’s MCAR test indicated data were missing completely at random (X^2^(163) = 163.62, *p* = 0.472).

Change scores (i.e., subtracting T1 scores from T3 scores) for total and subscale KINDL-R scores were the outcome measures. In the preregistration, T3 scores for QoL were taken as the outcome variables, but this was changed since doing so would not adequately answer our research question. The predictor variables were gender, SES, multilingualism, and educational support (environmental), Schlichting language tests and PPVT-III-NL (language, cognitive), CP (behavioural), and the SDQ-peers and -emotion subscales (behavioural). The emotions subscale was not mentioned in the preregistration, but was later considered necessary based on [8]. QoL at T1 was also included to account for each participant’s early QoL. As such, the results are interpretable as they are and are not influenced by differences in baseline QoL levels. SEM models included all predictors simultaneously. Model fit indices were not applicable, as the models were fully saturated and contained no latent variables.

Some predictor variables were adjusted for the purpose of the present analyses. Binary and ordinal variables were subject to effects coding (Gender, SES, multilingualism, and educational support). SES was originally ordinal, with three categories—low, medium, and high—but only 16 participants in this dataset had low SES. Participants with low and medium SES were therefore merged into one category, resulting in a binary variable for SES.

The language difficulties of the children included in our study range widely due to the early identification of presumed DLD. To substantiate our findings for the total group, analyses were also run on a subsample of 168 children with confirmed DLD. All figures and tables for these subgroup analyses are included in the Supplementary Materials.

## 3. Results

### 3.1. Differences in QoL over Time

**Figure 1:**
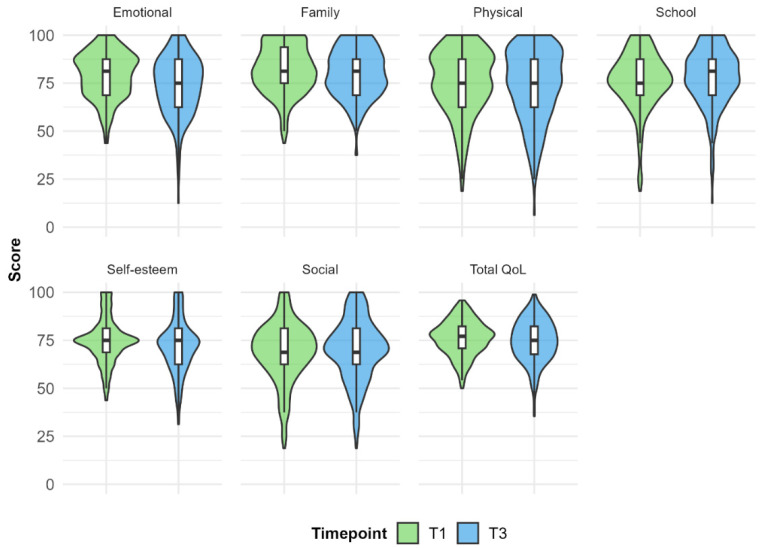
Violin plots of QoL scores at T1 and T3.

The distribution of QoL scores at T1 and T3 is visualised in Figure 1. Table 3 presents the QoL (KINDL scores) at T1 and T3, norm-referenced group comparisons, and paired *t*-test results. Children with DLD differed significantly from the norm group on all domains, except self-esteem at T3 and family well-being at both measurements. Participants scored lower than the norm-referenced group on all the significantly different dimensions, i.e., physical well-being, emotional well-being, social well-being, school, and overall QoL. Note that, although not significant, self-esteem scores were above the norm-referenced group at both timepoints. These findings were replicated in the subgroup analyses.

Paired sample *t*-tests were conducted to analyse differences in QoL between T1 and T3. Normality was visually assessed through histograms and QQ-plots, and no severe non-normality was observed. The T3 scores for emotional well-being, self-esteem, family, and overall QoL were significantly lower than at T1. Effect sizes (Cohen’s *d*) for those tests ranged from 0.120 to 0.386 and were small. No differences were found between the two time points for the other domains. Paired sample *t*-tests within the subgroup were only significant for emotional well-being (Cohen’s *d* = 0.384) and self-esteem (Cohen’s *d* = 0.206).

Table 4 displays the distribution regarding change over time, divided into improvement (T3 > T1), deterioration (T3 < T1), and stability (T3 = T1). Within most QoL domains, the group with stable QoL is relatively small (5–26%) compared to the children who improve (38–54%) and deteriorate (31–47%). This highlights the need to assess factors related to QoL across domains, not solely in domains that showed significant *t*-test results between T1 and T3 (Table 3). The distribution of changes in QoL in the subgroup with confirmed DLD was roughly the same as in the total sample, with the exception of social well-being. A larger percentage of children showed deterioration in this domain, namely 45% as opposed to 38% in the total sample.

### 3.2. Factors Associated with Change in QoL Between 4 and 9 Years

Table 5 presents the SEM results with change scores as the outcome variable. The models explained between 18.0% and 36.5% of the variance in QoL development between 4 and 9 years. The models identified significant predictors for change in emotional well-being and friendships, after *p*-value corrections for multiple testing. Multilingual status was positively related to change in emotional well-being, indicating that multilingual children’s emotional well-being changes less than that of monolinguals. Peer problems were negatively associated with social well-being, indicating that higher levels of peer problems were associated with fewer changes in social well-being. The associations between emotional well-being and multilingualism and between social well-being and peer problems were replicated in the subgroup analysis. Different from the total sample, peer problems were also negatively associated with emotional well-being and total QoL in the subgroup.

## 4. Discussion

This study investigated the QoL of children with DLD. We aimed to track QoL development between 4 and 9 years and identify (protective) factors associated with this change. The following discussion presents the results in the context of the guiding research questions.

### 4.1. Changes in the QoL of Children with Presumed and Confirmed DLD

To evaluate changes in QoL in this cohort, we compared QoL in children with DLD aged 4 to 9. The findings reveal notable group-level declines in emotional well-being and self-esteem, ultimately indicating an overall decline of QoL in our total sample of children with presumed DLD, but not in the subgroup with confirmed DLD. Thus, QoL is vulnerable in children with early language problems, irrespective of a later DLD diagnosis. The children’s QoL remains below that of the norm-referenced group at age 9, with the exceptions of self-esteem and family-related well-being (which were similar to the norm at both time points). This pattern mirrors the trajectory of language development in children with DLD: they follow a qualitatively similar developmental trajectory to TD children, but QoL levels are consistently lower. Given the similar trajectories observed between the groups with and without a confirmed DLD diagnosis, we will not distinguish between them in the discussion that follows.

The most pronounced changes were observed in self-esteem and emotional well-being, which both declined, although effect sizes for all significant reductions ranged from small to medium. These outcomes partially align with earlier research conducted by [8,15]. Although both studies’ criteria for the language-impaired group were more lenient than the criteria for DLD adhered to in this study (respectively, ≥1.25 and ≥1.5 SD below the mean on standardised language test(s)), Le et al. similarly indicate the vulnerability of general QoL in children with language problems. Eadie et al. identified a decline in the average QoL, albeit without a formal assessment of statistical significance. Drawing direct comparisons between our findings and those of Eadie et al. is limited by the use of different questionnaires in QoL, but some domains are similar. A discussion of these QoL domains will follow below.

Consistent with our findings, Eadie et al. reported lower emotional well-being at age 9 compared to age 4. These findings also coincide with studies on emotional functioning at the behavioural level, where children with DLD also experience difficulties with emotional abilities [30]. In contrast, Eadie et al. reported decreases in school, social, and physical functioning, which were not replicated in our cohort. The stability we observed in school and social functioning may reflect the children’s low starting point compared to the norm-referenced group, whereas the children with DLD in Eadie et al. only had school functioning below the norm at 4 years. Alternatively, these findings may result from the influence of the Dutch educational system, where children with DLD often receive tailored support in special education or additional assistance in mainstream schools. The support children receive may foster positive academic outcomes and school experiences, while also supporting social well-being through peer groups with similar communicative challenges and targeted language treatment. This may contribute to substantial improvements in communicative skills upon completion of special education, possibly reflected in the parental reports of relatively stable social and school functioning. Such suppositions warrant further investigation, given that recent research studying the impact of special and regular/inclusive education on the (psycho)social or academic outcomes of children with special educational needs is scarce, and previous research is inconclusive [36].

Interestingly, both [8] and our study report a decline in physical well-being scores, although the difference between T1 and T3 was not significant. The two questionnaires feature different items, yet both aim to assess children’s energy levels by inquiring about their endurance, strength, and experiences of physical pain, such as stomach aches and headaches. In both studies, the physical well-being of the norm-referenced group remains stable. The changes observed in our study may reflect children’s increased ability to communicate physical (dis)comfort, leading to lower parent-rated physical well-being. Alternatively, language, emotional, and/or social difficulties might negatively impact physical functioning, such as stomach/headaches or fatigue, affecting the ability to initiate or sustain physical activities.

Unlike measures used in previous studies on QoL in this population, the KINDL includes self-esteem and family well-being. In both the overall sample and the subgroup, self-esteem showed a significant decline between the ages of 4 and 9. For family well-being, a significant decline was only apparent in the overall sample. The implications of these findings warrant caution, particularly since scores in both domains remained within the normative range and effect sizes were small, suggesting that while the observed changes are noteworthy, they may not necessarily indicate concern. The absence of a decline in family well-being among children with confirmed DLD could reflect tighter family bonds, possibly due to the heightened parental support these children receive.

Overall, these findings underscore the vulnerability of the QoL of children with presumed DLD in the period between 4 and 9 years. Emotional well-being and self-esteem are particularly at risk, although challenges with self-esteem are also present among their TD peers.

### 4.2. Factors Associated with Change in QoL of Children with DLD

To elucidate the determinants of QoL change, this study explored the influence of cognitive, behavioural, and environmental factors, identifying direct effects of peer relations and multilingualism on QoL. We first interpret these effects, followed by a discussion of the domains for which no associated factors were found.

**Figure 2:**
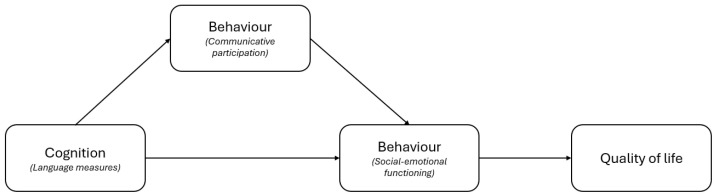
Proposed relationship between language, communicative participation, social–emotional functioning, and quality of life.

Difficulties in peer interaction emerged as a significant factor influencing changes in emotional and social well-being within the larger group, additionally impacting self-esteem and overall QoL among the subgroup with confirmed DLD. Prior research has shown that difficulties with initiating and maintaining peer relationships may persist and increase throughout childhood and adolescence [29,47]. These challenges are tied to early expressive language and reading abilities, with lower abilities predicting greater risk of continuing social problems [29,47]. Early negative experiences with peer interactions can adversely affect children’s self-esteem and social well-being, especially as the ability to establish and maintain peer relationships becomes increasingly important during childhood, when children are expected to navigate and sustain friendships independently. Taking into consideration the findings by [26] on the mediating role of CP between standardised language measures and well-being (SEF and QoL taken together) and the results of the present study, we propose a relationship between language (cognition), communicative participation (behaviour), social–emotional functioning (behaviour), and QoL as presented in Figure 2. Future research is necessary to confirm this hypothesis.

Multilingualism also exhibited direct associations with emotional well-being in both the total sample and the subgroup. Closer inspection revealed that this effect was primarily driven by monolingual children, whose emotional well-being declined over time, while multilingual children displayed relative stability. We think it is likely that the monolingual children’s trajectory reflects the broader trend within this population for two reasons. First, monolinguals form the larger group in our sample, with 82% of the children speaking one language, and only 18% speaking multiple languages. Second, the monolinguals’ trajectory is consistent with previous research into emotional difficulties in children with DLD [29]. Our discussion therefore focuses on the relative stability observed in the multilingual group, for which we suggest several tentative explanations. Importantly, this group was small, so our interpretation of this finding should be interpreted cautiously. One possibility is that compared to Dutch monolingual children, multilingual children may be raised in a different cultural environment. Such contexts can involve varying attitudes and values towards emotional behaviour (e.g., regulation and expression) and mental health [34,48], which may influence the parents’ responses to questions about emotional well-being. This raises an important question: do the observed differences between monolingual and multilingual children reflect variations in QoL, or do they reflect different response patterns? Our results can also be viewed as a confirmation of previous studies reporting that balanced bilinguals have equal or higher levels of well-being compared to monolinguals [35,36]. In this way, multilingualism, albeit with sufficient proficiency in the societal language, may protect against reduced QoL, also when children have DLD. Moreover, executive functions (EFs) may play a role in the relationship between multilingualism and emotional well-being. While EFs are a known weakness of children with DLD, evidence suggests an advantage in verbal and visuospatial working memory (WM) for bilingual children with DLD [49]. Since poor WM has been associated with emotion regulation problems and with mood or anxiety disorders [50], a hypothetical explanation for our finding is that the multilingual children in our sample benefited from a WM advantage, which may have protected their emotional well-being.

We did not identify factors associated with the other QoL domains (physical well-being, self-esteem, family, and school), nor for overall QoL. Several factors may contribute to this outcome. First, it is possible that our models did not adequately capture all relevant influences. An example of a cognitive aspect that has not been captured in this study is EF, which is linked to academic achievement and might affect school functioning. Additionally, other behavioural factors aside from peer relations may influence well-being. Difficulties with emotion understanding or expression were not included in our study, but may be related to QoL. Environmental factors, such as family circumstances, personality traits, and social support, could also play a role in shaping QoL development.

### 4.3. Limitations

This study’s limitations must be acknowledged. First, we relied on parent-reported measures of QoL, which may differ from children’s own perspectives [21]. However, at the first measurement, the children were unlikely to comprehend the questions and respond reliably due to their young age and language difficulties. Investigating change in children’s own perception of QoL between 4 and 9 years may therefore be challenging, although methods to measure the perspectives of (young) children with DLD, e.g., on their feelings or attitudes regarding communication, are used increasingly and may be useful for the development of QoL measures for this group (see e.g., SPAA-C by [51]). Second, the information on multilingualism is limited. We classified children as multilingual if one or both parents spoke to them in a language other than Dutch, but we did not collect data on the quality and quantity of the language input. A third limitation is our measure of SES, for two reasons. We based SES solely on the educational level of the best-educated parent. A composite measure, also including occupation and family income, would have provided a more sophisticated impression of participants’ SES. Moreover, our group of low SES participants was too small to include in the analyses. Fourth, although we examined several potentially influential factors for QoL, it was not possible to do this exhaustively. Ideally, a broader range of environmental and cognitive variables would have been included to provide a more comprehensive understanding of the determinants of QoL. For example, limited information was available about children’s home (literacy) environment and cultural background. In addition, assessments of behavioural EF tasks were not included, as it was not feasible to add them to the already extensive test battery of the DLDLD study. These limitations should be considered when interpreting the reported effects. Future research using self-reports, a well-established group of multilingual children, and a well-defined and broader range of cognitive and environmental measures will be necessary to understand the development in QoL of children with DLD.

## 5. Conclusions

The QoL of children with early language difficulties and those with confirmed DLD is vulnerable during (early) childhood. Their QoL is typically below the normative group, except for self-esteem and family well-being. On average, QoL is particularly fragile between 4 and 9 years, and emotional well-being deserves specific attention. Associations were found between change in QoL, early peer problems, and multilingual status. Children with early peer problems and monolingual children with DLD seem especially vulnerable to declines in emotional well-being and QoL in general. Peer problems had a larger effect on children with confirmed DLD, which may be related to the severity of their language difficulties. To establish the implications of these findings, further research is necessary, especially regarding a potential mediating role of peer problems in the relation between (expressive) language problems and QoL. Interpreting the effects of multilingual status is complex and should be done with caution. Future research should aim to replicate our findings on the association between multilingualism and QoL. Taken together, the results of this study underscore the importance of a multifactorial approach towards QoL. Clinicians should be aware of the potential long-term effects of early behavioural difficulties on the QoL of children with DLD and could explore ways to incorporate this during treatment.

## Figures and Tables

**Table 1:** Participant characteristics for the total group of children and the subgroup of children with confirmed DLD.

	N for Which Data Was Available	Total Sample	N for Which Data Was Available	Confirmed DLD
Male gender *n* (%)	296	215 (73)	168	117 (70)
Age (years) at T0, mean (sd)	272	3; 11 (0; 3)	157	3; 11 (0; 3)
Age (years) at T1, mean (sd)	296	4; 8 (0; 2)	168	4;8 (0; 2)
Age (years) at T3, mean (sd)	296	8; 10 (0; 6)	168	8; 10 (0; 6)
Multilingual *n* (%)	296	46 (16)	168	30 (18)
**SES ** ***n*** ** (%)**	293		168	
Low		16 (5)		9 (5)
Medium		87 (29)		55 (33)
High		190 (64)		104 (62)
Education regular/special T1 *n* (%)	261	143 (55)/118 (45)	157	39 (25)/118 (75)
NVIQ (SON-R 7 or 8), mean (sd)	259	102 (12.9)	152	101 (11.0)
**Language at T0, Q-scores, mean (sd)**				
Language comprehension (Schlichting)	253	91.2 (15.0)	147	88.8 (15.4)
Word comprehension (Peabody)	231	98.4 (12.4)	134	96.9 (12.9)
Word production (Schlichting)	261	88.2 (19.8)	151	83.7 (19.4)
Sentence production (Schlichting)	262	78.8 (9.9)	151	75.6 (8.8)

Note: SES was based on the parent with the highest level of education. Children categorised as “special education” attended cluster-2 education, a special type of education in the Netherlands, solely accessible to children with language difficulties. NVIQ was assessed with either the SON-R 2–7 or SON-R 2–8. To combine the tests, scores were first transformed to z-scores and then back to index scores.

**Table 2:** Overview of time points and measured variables.

T0 (3; 11 Years)	T1 (4; 8 Years)	T3 (8; 10 Years)
Word comprehension	Communicative participation	Quality of life
Language comprehension	Peer problems	Information about DLD diagnosis
Expressive vocabulary	Quality of life	
Expressive grammar	Educational support	
Non-verbal IQ		
Background information (SES/multilingualism)		

**Table 3:** Comparisons between QoL at T1 and T3.

KINDL Domains	T1 (4–5 Years)		T3 (8–9 Years)		t (291)	*p*	Cohen’s *d*
	DLD	Norm	DLD	Norm			
Physical well-being	75.47 (16.99) ***	80.2	73.92 (18.51) ***	80.5	1.143	0.254	0.067
Emotional well-being	79.32 (12.10) ***	83.0	73.86 (15.15) ***	82.3	6.591	<0.001***	0.386
Self-esteem	76.39 (14.89) *	73.6	72.23 (13.67)	70.8	3.404	<0.001***	0.199
Family	81.83 (12.23)	80.7	80.24 (12.36)	79.8	2.046	0.042 *	0.120
Social well-being	69.24 (15.40) ***	79.7	69.93 (16.22) ***	78.3	−0.969	0.333	−0.057
School	76.39 (14.89) ***	83.8	77.81 (14.98) ***	82.6	−1.431	0.152	−0.084
Total	76.23 (8.97) ***	80.0	74.67 (10.86) ***	79.0	2.491	0.013 *	0.120

Note. Group means were compared to the norm. Scores were lower than the norm-referenced group, except for self-esteem at T3 and family at T1 and T3. Asterisks indicate significant differences, with * indicating differences were significant at *p* < 0.05, and *** at *p* < 0.001. This applies to comparisons between the group mean and the norm-referenced group, as well as the paired samples *t*-tests.

**Table 4:** Number of participants who improved, deteriorated, or remained stable per KINDL score.

KINDL Score	Change							
	Improve			Deteriorate			Stable	
	*n*	%	Range	*n*	%	Range	*n*	%
Physical	126	43	6.25–68.75	125	43	−68.75–−6.25	41	14
Emotional	79	27	6.25–31.25	159	54	−81.25–−6.25	54	18
Self-esteem	90	31	6.25–37.5	126	43	−56.25–−6.25	76	26
Family	104	36	6.25–43.75	122	42	−37.5–−6.25	66	23
Social	122	42	6.25–56.25	110	38	−62.5–−6.25	60	21
School	137	47	6.25–56.25	116	40	−87.5–−6.25	39	13
Total	125	43	1.04–22.92	151	52	−42.71–−1.04	16	5

Note. Percentages may not sum to 100 due to rounding.

**Table 5:** Associations between cognitive, behavioural, and environmental factors and change in QoL between 4 and 9 years.

KINDL Domain	Variable(s)	Est.	SE	95%CI		*p*	*p* (Corrected)	Model R^2^
				LL	UL			
Physical	Word production	−0.169	0.085	−0.352	−0.019	0.029 *	0.136	0.365
	Emotional problems	−0.108	0.620	−2.530	−0.098	0.034 *	0.136	
Emotional	Multilingualism	0.164	3.188	2.421	10.148	0.001 **	0.009 **	0.181
	Emotional problems	−0.121	0.478	−1.878	−0.005	0.049 *	0.146	
	Peer problems	−0.149	0.582	−2.457	−0.174	0.024 *	0.095	
Self-esteem	Multilingualism	0.123	1.897	1.249	8.686	0.009 **	0.053	0.280
	Emotional problems	−0.116	0.474	−1.874	−0.018	0.046 *	0.183	
	Peer problems	−0.097	0.483	−1.843	0.050	0.063	0.190	
Family	No significant factor(s)							
Social	Language comprehension	0.142	0.069	0.025	0.296	0.021 *	0.068	0.342
	SES	−0.111	1.739	−7.368	−0.552	0.023 *	0.068	
	Multilingualism	0.081	2.35	−0.176	7.799	0.061	0.146	
	Peer problems	−0.224	0.655	−3.702	−1.133	<0.001 ***	0.001 ***	
School	No significant factor(s)							
Total QoL	Word production	−0.180	0.043	−0.175	−0.008	0.032 *	0.171	0.180
	Multilingualism	0.102	1.436	0.036	5.667	0.047 *	0.171	
	Peer problems	−0.114	0.385	−1.488	0.022	0.057	0.171	

Note: Only (almost) significant factors before Benjamini–Hochberg corrections are presented. Significance levels were determined as follows: * = *p* < 0.05, ** = *p* < 0.01, *** = *p* < 0.001.

## Data Availability

Restrictions apply to the dataset. The datasets presented in this article are not readily available because the data are part of an ongoing study. Requests to access the datasets should be directed to the corresponding author.

## Supplementary Materials

The following supporting information can be downloaded at: https://www.mdpi.com/article/10.3390/children13030418/s1, Questionnaire for Communicative Participation; Figure S1: Violin plots of QoL scores at T1 and T3; Table S1: Comparisons between QoL at T1 and T3; Table S2: Number of children who improved, deteriorated or remained stable per QoL score (measured with the KINDL). Table S3: Associations between cognitive, behavioural and environmental factors and change in QoL between 4 and 9 years.

## Abbreviations

The following abbreviations are used in this manuscript:

DLD	Developmental Language Disorder
TD	Typically developing
EF	Executive function
QoL	Quality of life
SEF	Social–emotional functioning
ToM	Theory of Mind
CP	Communicative participation
SES	Socio-economic status
