# Supplementary material for: Quality of Life in Children with Developmental Language Disorder
Source: Children (Basel). 2026 Mar 19;13(3):418. doi: 10.3390/children13030418 (PMC13025319; doi:10.3390/children13030418)
Supplement: Supplementary file 1 [file children-13-00418-s001.zip › children-4139858-supplementary.pdf]

Questionnaire for Communicative Participation

Question 1[1]

How severe is the limitation in communication of your child in comparison with peers?

Minimal limitation 1 2 3 4 5 6 7 8 9 10 Maximal limitation

Question 2

How worried are you about your child's speech-language development?

Not at all 1 2 3 4 5 6 7 8 9 10 Extremely worried

Question 3[2]

My child is able to communicate independently with us, their parent(s)/caregiver(s).

Not at all 1 2 3 4 5 6 7 8 9 10 Very well

Question 4

My child is able to communicate independently with their siblings or other members of the immediate family.

Not at all 1 2 3 4 5 6 7 8 9 10 Very well

Question 5

My child is able to communicate independently with other children.

Not at all 1 2 3 4 5 6 7 8 9 10 Very well

Question 6

My child is able to communicate independently with familiar adults.

Not at all 1 2 3 4 5 6 7 8 9 10 Very well

Question 7

My child is able to communicate independently with unfamiliar adults.

Not at all 1 2 3 4 5 6 7 8 9 10 Very well

Question 8

My child has social interaction with children outside of school hours.

Not at all 1 2 3 4 5 6 7 8 9 10 Very often

---

[1] Questions 1 and 2 were removed from analyses due to low internal consistency.

[2] Parent(s)/Caregiver(s) were given the following definition for independent communication: A child is able to communicate independently if they can communicate their message without the help of a parent, caregiver or other adult.

Question 9

My child has friendships with children.

Not at all 1 2 3 4 5 6 7 8 9 10 Many

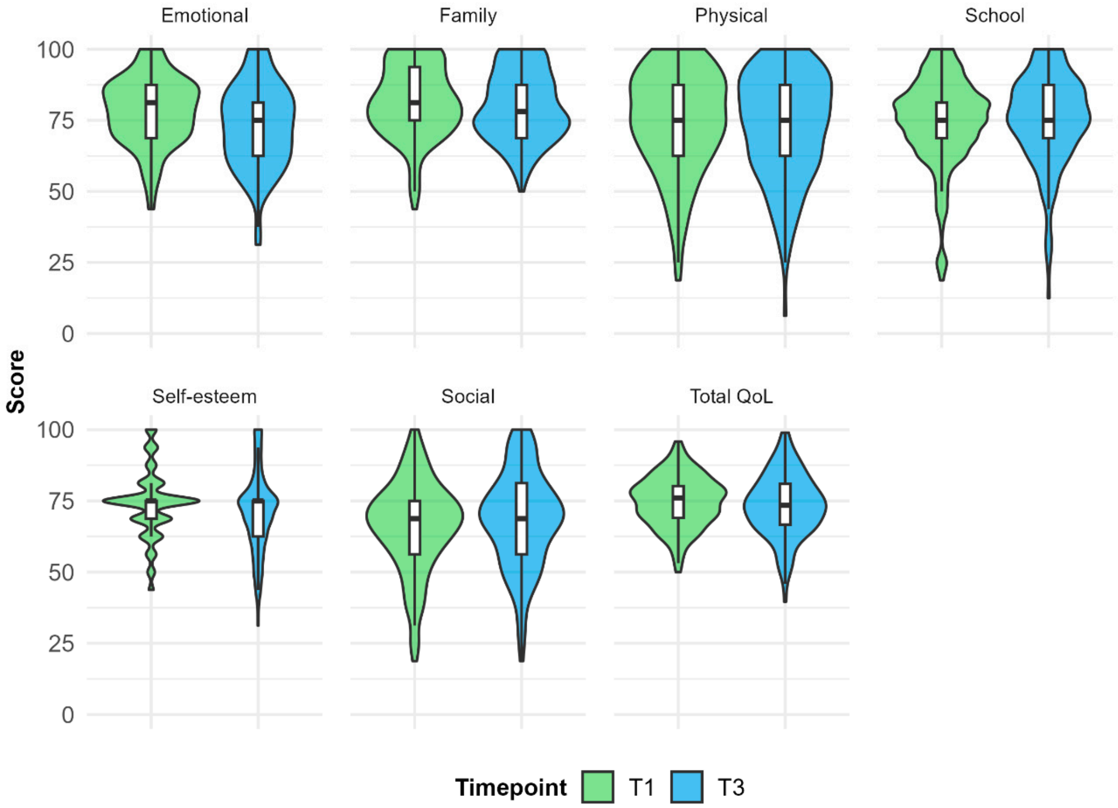

Figure S1. *Violin plots of QoL scores at T1 and T3 – confirmed DLD.*

Table S1. *Comparisons between QoL at T1 and T3 – confirmed DLD.*

KINDL domains	T1 (4-5 years)		T3 (8-9 years)		*t*(165)	*p*	Cohen's *d*
	M (SD)	*Norm*	*M (SD)*	*Norm*			
Physical well-being	74.36 (17.97)***	80.2	72.81 (18.54)***	80.5	.875	.383	.068
Emotional well-being	78.24 (11.91)***	83.0	73.07 (14.59)***	82.3	4.954	**<.001*****	.384
Self-esteem	73.91 (11.35)	73.6	71.24 (13.83)	70.8	2.657	**.009****	.206
Family	81.33 (12.44)	80.7	79.80 (12.06)	79.8	1.445	.150	.112
Social well-being	66.30 (15.73)***	79.7	68.38 (16.58)***	78.3	-1.484	.140	-.115
School	74.62 (15.67)***	83.8	75.67 (15.85)***	82.6	-.660	.510	-.051
Total QoL	74.79 (8.88)***	80.0	73.49 (10.91)***	79.0	1.752	.082	.136

Note. Group means were compared to the norm. Scores were lower than the norm-referenced group, except for self-esteem at T3 and family at T1 and T3. Asterisks indicate significant differences, with * indicating differences were significant at p<.05, and *** at p<.001. This applies to comparisons between the group mean and the norm-referenced group, as well as the paired samples t-tests.

Table S2. *Number of children who improved, deteriorated or remained stable per QoL score (measured with the KINDL).*

KINDL score	Change							
	Improve			Deteriorate			Stable	
	n	%	Range	n	%	Range	n	%
Physical	72	43	6.25 – 68.75	74	45	-68.75 - -6.25	20	12
Emotional	45	27	6.25 – 31.25	89	54	-43.75 – -6.25	32	19
Self-esteem	46	28	6.25 – 31.25	74	45	-56.25 - -6.25	46	28
Family	58	38	6.25 – 31.25	69	42	-37.5 – - 6.25	39	23
Social	64	39	6.25 – 56.25	75	45	-43.75 - -6.25	27	16
School	78	47	6.25 – 43.75	67	40	-87.5 - -6.25	21	13
Total	70	42	1.04 – 20.8	85	51	-39.58 - -1.04	11	7

Note. Percentages may not sum exactly to 100 due to rounding.

Table S3. *Associations between cognitive, behavioural and environmental factors and change in QoL between 4 and 9 years – confirmed DLD.*

KINDL domain	Variable(s)	Est.	SE	95%CI		*p*	*p* (corrected)	Model R^2
				LL	UL			
Physical	TBQ	.156	.109	.011	.438	.039*	.118	.424
	Education	.182	4.144	.958	17.201	.028*	.114	
	SES	.122	2.870	-.060	11.192	.052.	.126	
	Peer problems	-.144	.871	-3.697	-.284	.022*	.114	
Emotional	Word comprehension	-.241	.117	-.486	-.026	.029*	.087	.260
	Multilingualism	.222	2.373	3.278	12.579	<.001***	**.005****	
	Peer problems	-.230	.616	-3.175	-.762	.001**	**.006****	
Self-esteem	Multilingualism	.161	2.283	1.201	10.152	.013*	.078	.222
	Peer problems	-.140	.543	-2.245	.118	.029*	.118	
Family	No significant factor(s)							
Social	Peer problems	-.305	.803	-4.943	-1.805	<.001***	**<.001*****	.378
School	No significant factor(s)							
Total QoL	Multilingualism	.145	1.722	.323	7.072	.032*	.127	.196
	Peer problems	-.187	.396	-1.916	-.365	.004**	**.024***	

Note. Only (almost) significant factors before Benjamini-Hochberg corrections are presented. Significance levels were determined as follows: * = p<.05, ** = p<.01, *** = p<.001.
